# Multistate Outbreak of Listeriosis Associated with Packaged Leafy Green Salads, United States and Canada, 2015–2016

**DOI:** 10.3201/eid2508.180761

**Published:** 2019-08

**Authors:** Julie L. Self, Amanda Conrad, Steven Stroika, Alikeh Jackson, Laura Whitlock, Kelly A. Jackson, Jennifer Beal, Allison Wellman, Marianne K. Fatica, Sally Bidol, Paula Pennell Huth, Meghan Hamel, Kristyn Franklin, Lorelee Tschetter, Christine Kopko, Penelope Kirsch, Matthew E. Wise, Colin Basler

**Affiliations:** Centers for Disease Control and Prevention, Atlanta, Georgia, USA (J.L. Self, A. Conrad, S. Stroika, L. Whitlock, K.A. Jackson, M.E. Wise, C. Basler);; US Food and Drug Administration, College Park, Maryland, USA (A. Jackson, J. Beal, A. Wellman, M.K. Fatica);; Michigan Department of Health and Human Services, Lansing, Michigan, USA (S. Bidol);; New York State Department of Health, New York, New York, USA (P. Pennell Huth);; Public Health Agency of Canada, Ottawa, Ontario, Canada (M. Hamel, K. Franklin, L. Tschetter);; Canadian Food Inspection Agency, Ottawa (C. Kopko, P. Kirsch)

**Keywords:** listeriosis, Listeria monocytogenes, bacteria, foodborne infections, food safety, packaged leafy green salads, lettuce, outbreak, produce, United States, Canada

## Abstract

We investigated an outbreak of listeriosis detected by whole-genome multilocus sequence typing and associated with packaged leafy green salads. Nineteen cases were identified in the United States during July 5, 2015–January 31, 2016; isolates from case-patients were closely related (median difference 3 alleles, range 0–16 alleles). Of 16 case-patients interviewed, all reported salad consumption. Of 9 case-patients who recalled brand information, all reported brands processed at a common US facility. The Public Health Agency of Canada simultaneously investigated 14 cases of listeriosis associated with this outbreak. Isolates from the processing facility, packaged leafy green salads, and 9 case-patients from Canada were closely related to US clinical isolates (median difference 3 alleles, range 0–16 alleles). This investigation led to a recall of packaged leafy green salads made at the processing facility. Additional research is needed to identify best practices and effective policies to reduce the likelihood of *Listeria monocytogenes* contamination of fresh produce.

Invasive *Listeria monocytogenes* infections (listeriosis) are the third leading cause of death from foodborne illness in the United States and cause an estimated 1,500 infections, 1,400 hospitalizations, and 250 deaths each year (*1*). Although incidence of listeriosis is lower than for many foodborne illnesses, it often results in severe illnesses, including sepsis and meningitis, and is associated with a high case-fatality rate and fetal loss in pregnant women (*2*–*4*). Populations at highest risk for invasive listeriosis include elderly persons, immunocompromised persons, and pregnant women and their newborns (*5*). Listeriosis can have a long incubation period (median 11 days, range 0–70 days) between exposure and symptom onset (*5**,**6*).

*L. monocytogenes* was first recognized as a foodborne pathogen after an outbreak in Canada during 1981 that was linked to cabbage in coleslaw (*7*). Outbreaks of listeriosis in the United States have historically been associated with ready-to-eat delicatessen meats and dairy products, but have more recently been associated with fresh produce, including sprouts, celery, cantaloupe, stone fruit, and caramel apples (*5**,**8**,**9*).

In October 2015, PulseNet USA (https://www.cdc.gov/pulsenet/index.html), the national molecular subtyping network for foodborne disease surveillance, identified a cluster of 8 clinical *L. monocytogenes* isolates from 6 states that were closely related genetically to one another by whole-genome multilocus sequence typing (wgMLST) (median difference 5 alleles, range 0–12 alleles); the Centers for Disease Control and Prevention (CDC; Atlanta, GA, USA) initiated a cluster investigation. During a weekly foodborne cluster investigation call on January 13, 2016, CDC informed the Public Health Agency of Canada (PHAC) of the cluster investigation, which had increased to include 13 cases of listeriosis. PHAC informed CDC of a cluster of 6 cases of listeriosis in Canada that had a pulsed-field gel electrophoresis (PFGE) pattern combination indistinguishable from that of the US cases. The United States and Canada conducted collaborative investigations to determine the source of the outbreak.

## Methods

### Investigation in the United States

#### Epidemiologic Investigation

We defined a case as laboratory-confirmed invasive listeriosis in which *L. monocytogenes* was isolated from a normally sterile site (e.g., blood or cerebrospinal fluid) or from products of conception (e.g., placental or fetal tissue). Isolates were indistinguishable from the outbreak PFGE pattern combination and closely related genetically to the outbreak clade by wgMLST (0–16 alleles) (*10*). Isolation dates were July 5, 2015–January 31, 2016.

In the United States, state and local health departments attempt to interview all patients with listeriosis (or their proxies) by using the *Listeria* Initiative (LI) questionnaire, which collects standard clinical, laboratory, and demographic information, as well as data for food exposures in the 28 days before illness onset (*11*). At the time of this investigation, the LI questionnaire included questions on 44 foods considered to have higher risk for *L. monocytogenes* contamination on the basis of previous outbreaks, case–control studies, and expert opinion (*12**,**13*). Questions about produce consumption were limited to melons, sprouts, fruit salad, coleslaw, and other ready-to-eat delicatessen-style salads (*11**,**12*). We conducted case–case comparisons by using LI questionnaire data to identify common food exposures among patients and generate hypotheses about possible outbreak food vehicles. During cluster investigations, case–case comparisons can be used in lieu of traditional but time- and resource-intensive case–control comparisons by comparing exposures reported for cluster-associated cases with those reported for non–cluster-associated (sporadic) cases (*14**,**15*).

After initial LI questionnaire case–case comparisons failed to identify a possible food vehicle for the outbreak, we developed a supplemental questionnaire that was implemented on October 8, 2015, to collect information about foods previously associated with the outbreak PFGE pattern, including cheeses, leafy green salads, stone fruit, and caramel apples. On December 9, 2015, we began conducting single-interviewer, semistructured, open-ended interviews by using an iterative approach to identify potential food exposures not included in the previous questionnaires and obtain more complete details on food products, purchase locations, and brands (*16**,**17*). During open-ended interviews, we asked about all foods consumed in the month before illness onset. When available, we reviewed grocery receipts, shopper card records, school and residential facility menus, and detailed diet logs to verify exposure details and purchase dates. Data were collected as part of the response to a public health emergency and did not meet the definition of research as provided by 45 Code of Federal Regulations 46.102(d).

#### Regulatory Investigation

The Ohio Department of Agriculture (ODA) routinely collects and tests retail foods for *L. monocytogenes* and other foodborne pathogens. Retail food samples can provide major clues during outbreak investigations if molecular subtyping identifies isolates as being closely related genetically to clinical isolates. US Food and Drug Administration (FDA) officials inspected and reviewed records from facilities linked to any *L. monocytogenes* isolated from food.

#### Laboratory Investigation

Clinical and food samples that yielded *L. monocytogenes* were subtyped by PFGE at state public health laboratories and FDA field laboratories by using *Asc*I and *Apa*I restriction endonucleases according to PulseNet standardized protocols (*18*). We performed whole-genome sequencing (WGS) for all isolates and analyzed results in BioNumerics version 7.5 (Applied Maths, http://www.applied-maths.com) by using wgMLST and the Lyve-SET pipeline (*10*). Sequence data were uploaded to the National Center for Biotechnology Information database (https://www.ncbi.nlm.nih.gov) for sequencing analysis.

### Investigation in Canada

In Canada, a case was defined as illness in a resident or visitor to Canada with laboratory-confirmed listeriosis and isolates having any of 3 outbreak PFGE pattern combinations and symptom onset on or after May 1, 2015. The primary PFGE pattern combination in the outbreak in Canada was indistinguishable from that of the outbreak in the United States; 2 additional PFGE pattern combinations (patterns A and B), were included for the outbreak in Canada. Initial interviews were completed by either the national Enhanced Listeriosis Surveillance Program questionnaire or provincial listeriosis questionnaires to gather food exposures during the 4 weeks before illness onset. After leafy green salads were identified as a suspected vehicle, a coordinated, centralized interviewing approach was used to reinterview patients (or proxies) who had isolates indistinguishable from those with the primary outbreak PFGE pattern.

The Canadian Food Inspection Agency (CFIA, Ottawa, Ontario, Canada) used a targeted retail sampling plan to obtain packaged leafy green salads made at a suspected processing facility from stores in Ontario and Nova Scotia during January 18–19, 2016. The targeted sampling plan included multiple salad varieties and manufacturer’s code combinations from multiple processing lines that were processed during January 3–14.

Provincial laboratories performed PFGE typing of clinical isolates, and CFIA performed PFGE typing on food isolates. PFGE patterns were uploaded to the PulseNet Canada national database (https://www.canada.ca/en/public-health/programs/pulsenet-canada.html) for national designation by the National Microbiology Laboratory (Winnipeg, Manitoba, Canada). WGS was completed by the National Microbiology Laboratory, Public Health Ontario Laboratory (Toronto, Ontario, Canada), and CFIA on all Canadian clinical and food isolates by using single nucleotide polymorphisms (SNPs) with the SNVPhyl pipeline (*19*) and wgMLST in BioNumerics version 7.5. We compared isolates from Canada with those from the United States by using PFGE and WGS.

## Results

### Investigation in the United States

#### Epidemiologic Investigation

**Figure 1:**
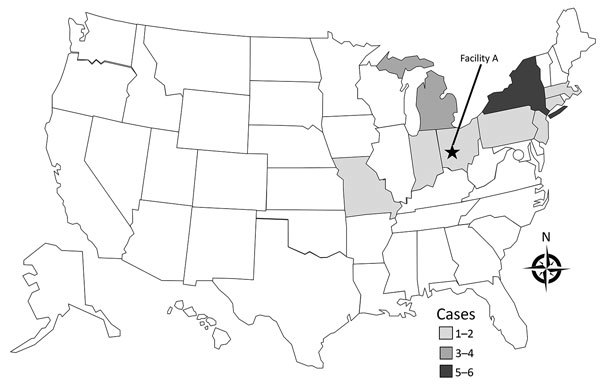
Outbreak-related cases of listeriosis (n = 19) in the United States by state of residence, July 5, 2015–January 31, 2016.

**Figure 2:**
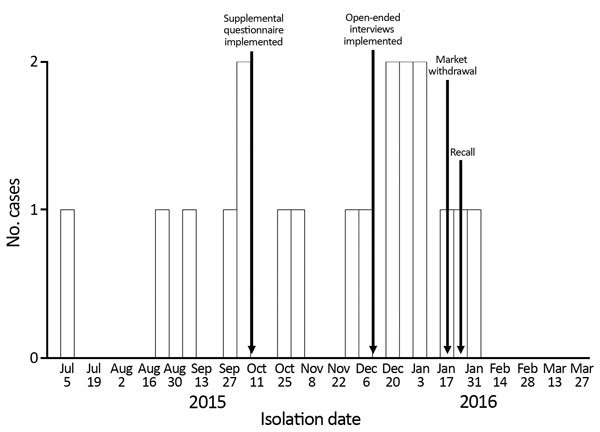
Timeline of *Listeria monocytogenes* isolation for 19 US patients during outbreak associated with packaged leafy green salads, July 5, 2015–January 31, 2016.

We identified 19 cases of listeriosis in 9 states: Connecticut (1), Indiana (1), Massachusetts (1), Michigan (4), Montana (2), New Jersey (1), New York (6), Ohio (2), and Pennsylvania (1) (Figure 1). All patient isolates were serotype 4b, indistinguishable by PFGE pattern combination, 7-gene sequence type (ST) 382, and closely related genetically to one another by wgMLST, differing by a median of 3 alleles (range 0–16 alleles) (*20*). Illness onset dates were July 5, 2015–January 31, 2016 (Figure 2). Of the 8 cases initially detected, 5 were later excluded by using this case definition because the isolates were different by >16 alleles and had isolation dates before July 5, 2015.

All 19 patients were hospitalized; listeriosis contributed to 1 (5%) death. Case-patients had a median age of 64 (range 3–83) years; 14 (74%) were female. One case of listeriosis in a pregnant woman resulted in a preterm live birth. Meningitis developed in 1 otherwise healthy child with listeriosis (Table 1).

**Table 1:** Characteristics of 19 patients with outbreak-related listeriosis associated with packaged leafy green salads, United States, July 5, 2015–January 31, 2016*

Characteristic	Value
Age, y, median (range)	64 (3–83)
Female sex	14 (74)
Pregnancy-associated cases†	1 (5)
Hospitalization	19 (100)
Death	1 (5)
*Listeria monocytogenes* culture site	
Blood	14 (74)
Cerebrospinal fluid	3 (16)
Placenta	1 (5)
Hip	1 (5)

*Values are no. (%) unless otherwise indicated.  †A pregnancy-associated case was defined as an illness in a pregnant woman or infant <28 d of age. Cases involving mother–infant pairs are counted as a single case.

No exposures on the standard LI questionnaire were commonly reported by case-patients in the cluster, and case–case comparisons did not generate any potential food items. Five case-patients completed supplemental interviews, and 12 case-patients completed iterative, open-ended interviews. One patient refused to be reinterviewed but provided a detailed dietary log for analysis. Ultimately, data on leafy green vegetable consumption was available for 16 (84%) of 19 patients; all 16 reported consuming leafy green salads, including 81% (13/16) reporting romaine lettuce and 71% (10/14) reporting spinach. Of those specifically asked, 93% (13 of 14) reported consuming packaged leafy green salads in the 28 days before illness onset. Nine case-patients recalled brand information; 2 packaged leafy green salad brands were reported that we determined came from a single processing facility in the United States (Table 2). Five case-patients were able to provide receipts or shopper card records to confirm purchase dates, locations, and brands. School or residential facility menus and invoices suggested potential exposure to leafy green salads for 2 additional case-patients who could not be interviewed.

**Table 2:** Consumption of leafy green salads for patients with outbreak-associated listeriosis, United States, July 5, 2015–January 31, 2016

Leafy green salads consumed	No. reported/no. responses (%)
Any leafy green salad	16/16 (100)
Romaine	13/16 (81)
Spinach	10/14 (71)
Packaged salad	13/14 (93)
Packaged salad brand processed at facility A	9/9 (100)

#### Regulatory Investigation

On January 14, 2016, PulseNet staff analyzed sequence data from *L. monocytogenes* isolated from a packaged leafy green salad that was collected by ODA during routine retail sampling and found that it was closely related by wgMLST to clinical isolates from case-patients (median 3 alleles, range 0–16 alleles). On the basis of these laboratory results, in combination with preliminary epidemiologic data suggesting a link to leafy green salads, on January 16, 2016, FDA initiated an inspection of the facility that processed the packaged leafy green salad.

During the facility inspection, FDA investigators observed factors that might contribute to *L. monocytogenes* contamination, including failure to collect environmental samples from food contact surfaces, collection of environmental samples only before production, and failure to conduct follow-up investigations beyond the standard practices after identifying *Listeria* spp. from environmental samples over a period of 19 months (*21*). Review of records indicated that the sampling program of the facility had yielded *Listeria* spp. 11 times during July 21, 2014–January 7, 2016. Finished product, including a Caesar salad kit, and in-line samples of romaine lettuce from a production line collected by FDA from the facility on January 16, 2016, yielded *L. monocytogenes* (Table 3). Results from the Caesar salad kit were confirmed positive for *L. monocytogenes* on January 26, 2016, and those for in-line romaine lettuce were confirmed positive for *L. monocytogenes* on January 28. *L. monocytogenes* was isolated on February 9, 2016, from an open container of packaged leafy green salad collected from the home of 1 case-patient; the salad had been processed at the same facility in the United States. Five isolates identified from the processing facility and 1 leftover product from the home of a patient were closely related by wgMLST to clinical isolates and the retail isolate (median 3 alleles, range 0–16 alleles).

**Table 3:** Food isolates yielding the outbreak strain of *Listeria monocytogenes*, United States and Canada*

Location of packaged salad sample collection	Packaged salad product	Collected by
Retail store in Ohio	Field greens	Ohio Department of Agriculture
US processing facility	Caesar	FDA
US processing facility	Romaine (in-process)	FDA
Patient’s home	Romaine	Ohio Department of Health
Retail store in Canada	Chopped Romaine	CFIA
Retail store in Canada	Caesar Salad Kit	CFIA
Retail store in Canada	Caesar Salad Kit	CFIA
Retail store in Canada	Colorful Coleslaw†	CFIA

*CFIA, Canadian Food Inspection Agency; FDA, Food and Drug Administration. †Yielded *L. monocytogenes* with secondary pulsed-field gel electrophoresis pattern B.

The inspection findings, the well-documented ability of *Listeria* species to persist in food processing/manufacturing environments, and the span of clinical cases over a period of months suggested that the source of the contamination was likely the processing/manufacturing environment, rather than a harvest site. As a result, no traceback to source farms was performed. After the FDA inspection, the processing facility implemented corrective actions.

### Epidemiologic Investigation in Canada

PHAC identified 14 cases of listeriosis in 5 eastern provinces: Ontario (9), Quebec (2), New Brunswick (1), Prince Edward Island (1), and Newfoundland and Labrador (1). Illness onset dates were May 7, 2015–February 23, 2016. Ten case-patients matched the primary outbreak PFGE pattern, and 4 matched secondary PFGE pattern A. Nine (90%) of 10 isolates from patients in Canada with the primary PFGE pattern were closely related genetically to one another (0–5 SNPs) and would have met the US case definition; 1 patient from Canada with the primary PFGE pattern was not closely related to the others by WGS (>35 SNPs from outbreak cases). Isolates with secondary PFGE pattern A were not closely related genetically to one another and were genetically distinct from isolates with the primary PFGE pattern. All case-patients in Canada were hospitalized, and 3 (21%) died, but it was not determined whether listeriosis contributed to the deaths.

Detailed food exposure information was collected for all 10 case-patients who had the primary outbreak PFGE pattern. Eight (80%) reported consuming packaged leafy green salads and salad kits before illness onset. Brand information was available for 4 case-patients, and all reported or provided a purchase history that included brands made at the same US processing facility. One case-patient with secondary PFGE pattern A was interviewed by using the focused questionnaire and reported consuming coleslaw but was unable to recall the brand or product details.

### Regulatory Investigation in Canada

CFIA collected and tested 137 packaged leafy green salads at the retail level, representing 45 unique product type and lot code combinations processed at the same US processing facility during January 3–14, 2016. Packaged leafy green salads from 4 unique product types and lot code combinations yielded *L. monocytogenes*; these products included 3 varieties of packaged salad (Chopped Romaine, Caesar Salad Kit, and Colorful Coleslaw), 4 production dates (January 9–14, 2016), and 3 production lines (Table 3). Three of these varieties yielded the primary PFGE pattern that was indistinguishable from the US outbreak strain. The isolate from Colorful Coleslaw, which was distributed only in Canada, had secondary PFGE pattern B, which was not closely related by wgMLST to other clinical or food isolates. WGS showed that all food isolates from Canada that had the primary outbreak PFGE pattern were closely related to one another, to the 9 closely related clinical isolates from Canada that had the primary PFGE pattern, and to the US clinical and food isolates (range 0–16 SNPs) (*20*).

### Product Actions and Public Reporting

On the basis of the epidemiologic data and the ODA retail surveillance sample of packaged salad that yielded the outbreak strain of *L. monocytogenes,* on January 21, 2016, the US processing facility voluntarily stopped production of all packaged leafy green salads because of possible *L. monocytogenes* contamination. On January 22, the facility issued a market withdrawal of products manufactured at that location, including 22 varieties of packaged salad sold under 6 brands in the United States and 2 brands in Canada, both organic and nonorganic brands. These products were distributed to >23 states in the eastern and midwestern United States and eastern regions of Canada (*22*,*23*). CDC, FDA, PHAC, and CFIA issued communications advising the public how to identify affected products by the manufacturing code found on the package and advised consumers and retailers to discard affected products (*22**–**25*). Consumers and retailers were also advised to thoroughly wash and sanitize anything that might have come in contact with the affected products, including refrigerator drawers and shelves, reusable grocery bags, food storage containers, countertops, and food preparation tools (*22*,*24*,*26*). On January 27, after receiving notification from FDA that samples from packaged leafy green salads collected during the facility inspection were confirmed positive for *L. monocytogenes*, the firm issued a voluntary recall of all packaged salad products made at the US processing facility with information for consumers to identify the recalled products and brands (*27*).

## Discussion

The combination of epidemiology, retail food sampling, environmental investigation, and laboratory data confirmed packaged leafy green salads from a single processing facility in the United States as the source of this listeriosis outbreak. Observations from the facility inspection suggest that the environmental sampling plan at the facility might have limited the ability of the facility to identify *L. monocytogenes* contamination or harborage, which might have contributed to food contamination. Production was halted at the facility for 4 months, during which time the facility conducted testing and a root cause investigation (*28*). The recall and suspension of operations cost the firm an estimated $25.5 million (*28*).

Several unique aspects of this investigation were essential in detecting this outbreak and identifying the food vehicle, which likely prevented additional illnesses and deaths. First, wgMLST was instrumental in distinguishing the isolates in this cluster from other *L. monocytogenes* isolates with the same common PFGE pattern, which has previously been isolated from multiple foods (*29**,**30*). PFGE provided a standard typing scheme to facilitate interagency communication. However, the number of cases with this PFGE pattern was not above the baseline number of expected cases, and the cluster would not have been detected and could not be defined by PFGE alone. wgMLST offers greater specificity than PFGE subtyping methods and demonstrated that clinical and packaged leafy green salad isolates were closely related genetically.

Second, use of single-interviewer, open-ended iterative interviews was essential for identifying this novel vehicle. Previous foodborne outbreak investigations have identified outbreak food vehicles by using this approach after standard interview procedures were unable to identify a likely source of infection (*16**,**17*). At the time of the outbreak, the LI questionnaire did not include questions about leafy green salads, and produce questions were limited to melons, sprouts, fruit salad, coleslaw, and other ready-to-eat delicatessen-style salads. The supplemental questionnaire developed for this investigation included questions about leafy green vegetables, but data collected did not identify a specific food item or brand. In April 2016, health departments in the United States began using an updated LI questionnaire that included additional questions about produce exposures, including leafy green salads and other foods identified in recent outbreaks or recalls. In addition, institutional menus, invoices, shopper card records, and personal dietary logs can be helpful in documenting exposure details that might otherwise be unavailable and identifying new vehicles.

Third, routine retail sampling by ODA and sequencing by the state laboratory provided a molecular association between clinical isolates and a specific food product that likely would not have been made in the absence of wgMLST. Packaged leafy green salads were the leading hypothesis in the epidemiologic investigation, but the molecular relationship between the retail food isolate and clinical isolates strengthened the early epidemiologic evidence for a link to packaged leafy green salads and provided evidence needed to identify the facility and processing environment for the investigation to move forward, setting the stage for the market withdrawal and subsequent recall.

Fourth, collaboration between investigators in the United States and Canada strengthened the investigation and likely contributed to a more timely and comprehensive market withdrawal and recall. Frequent, open communication between public health and regulatory partners from both countries ensured that each party had relevant information about suspected food vehicles and laboratory findings. Extensive retail product sampling by CFIA demonstrated that the contamination of products from the implicated facility was not restricted to a single product, production line, or production day, suggesting potentially widespread contamination, and helped determine the scope of the market withdrawal and recall. WGS was also helpful in demonstrating that clinical and food isolates from Canada and the United States were closely related genetically.

Leafy green salads were one of several suspected foods identified during investigation of an outbreak of listeriosis in a hospital in Boston in 1979, and food safety research has demonstrated *L. monocytogenes* is capable of contaminating leafy green salads and ready-to-eat salads (*7**,**31**–**39*). However, outbreaks of listeriosis were rarely linked to produce items until outbreaks in the past decade linked to sprouts, celery, cantaloupe, stone fruit, and caramel apples. Reasons for the emergence of fresh produce as a vehicle for *L. monocytogenes* are unclear and might be based on increased contamination of fresh produce or improved detection. One hypothesis is that increased postharvest processing and technologies that enable increased shelf life of many products, including fresh produce, create an opportunity for proliferation of *L. monocytogenes*. Unlike many other foodborne pathogens, *L. monocytogenes* can grow at cooler temperatures (*40*). Studies with lettuce have demonstrated that although most foodborne pathogens on lettuce decrease in number under proper storage conditions, *L. monocytogenes* can multiply (*31**,**41*). Other plausible explanations for an increase in identification of *L. monocytogenes* associated with produce outbreaks include improved detection of clusters because of advanced molecular techniques, such as WGS, and improved outbreak investigation techniques, such as availability of shopper card data.

Unlike most other foodborne pathogens, *L. monocytogenes* bacteria have the potential to grow in cold processing/manufacturing environments and form biofilms. Therefore, additional steps might be necessary to reduce the risk for *L. monocytogenes* contamination (*31**,**42**,**43*). For this outbreak, several factors suggest persistent contamination at the processing/manufacturing facility: distribution of illnesses over many months, facility records indicating the presence of *Listeria* spp. throughout a 19-month period, and CFIA retail sampling results indicating contamination on multiple days and manufacturing lines. A single case of listeriosis in the United States in 2013 was linked by wgMLST to an isolate from packaged leafy green salad produced at the same facility in the United States implicated in this investigation. The isolate from 2013 was not closely related by wgMLST (difference >1,400 alleles) to the isolates from this outbreak (*44*), but these findings suggest possible long-term, ongoing issues with *L. monocytogenes* contamination in this facility. In 2017, FDA published draft guidance for control of *L. monocytogenes* in ready-to-eat foods (*45*).

Vegetables and fruits are fundamental components of a healthy diet, and the US Department of Agriculture recommends that half of a person’s diet be composed of fruits and vegetables (*46*). CDC and FDA recommend that consumers follow general food safety practices for fruits and vegetables, including leafy green salads: properly refrigerating and separating from other foods such as raw meat and seafood, discarding products that are spoiled or have been recalled, and washing thoroughly to remove surface contamination unless the packaging indicates products are prewashed or ready-to-eat (*47**,**48*). However, the effectiveness of washing produce to reduce contamination varies by produce type, prior storage temperature, and washing method (*39**,**49**,**50*). Additional research should focus on identifying best practices and effective policies to reduce the likelihood of *L. monocytogenes* contamination of fresh produce, especially as technological innovations enable increased shelf life of packaged leafy green salads and other produce.
